# Supercritical Carbon Dioxide Treatment of Porous Silicon Increases Biocompatibility with Cardiomyocytes

**DOI:** 10.3390/ijms221910709

**Published:** 2021-10-02

**Authors:** David Jui-Yang Feng, Hung-Yin Lin, James L. Thomas, Hsing-Yu Wang, Chien-Yu Lin, Chen-Yuan Chen, Kai-Hsi Liu, Mei-Hwa Lee

**Affiliations:** 1 Department of Electrical Engineering, National University of Kaohsiung, Kaohsiung 81148, Taiwan; w612115@gmail.com (H.-Y.W.); liukaihsi@gmail.com (K.-H.L.); 2 Department of Chemical and Materials Engineering, National University of Kaohsiung, Kaohsiung 81148, Taiwan; linhy@ntu.edu.tw (H.-Y.L.); oaqoaq557788@gmail.com (C.-Y.L.); tp65p4m0@gmail.com (C.-Y.C.); 3 Department of Physics and Astronomy, University of New Mexico, Albuquerque, NM 87131, USA; jthomas@unm.edu; 4 Department of Internal Medicine, Division of Cardiology, Zuoying Branch of Kaohsiung Armed Forces General Hospital, Kaohsiung 81342, Taiwan; 5 Department of Materials Science and Engineering, I-Shou University, Kaohsiung 84001, Taiwan

**Keywords:** porous silicon, surface treatment, cardiomyocyte, biocompatibility, angiogenesis

## Abstract

Porous silicon is of current interest for cardiac tissue engineering applications. While porous silicon is considered to be a biocompatible material, it is important to assess whether post-etching surface treatments can further improve biocompatibility and perhaps modify cellular behavior in desirable ways. In this work, porous silicon was formed by electrochemically etching with hydrofluoric acid, and was then treated with oxygen plasma or supercritical carbon dioxide (scCO_2_). These processes yielded porous silicon with a thickness of around 4 μm. The different post-etch treatments gave surfaces that differed greatly in hydrophilicity: oxygen plasma-treated porous silicon had a highly hydrophilic surface, while scCO_2_ gave a more hydrophobic surface. The viabilities of H9c2 cardiomyocytes grown on etched surfaces with and without these two post-etch treatments was examined; viability was found to be highest on porous silicon treated with scCO_2_. Most significantly, the expression of some key genes in the angiogenesis pathway was strongly elevated in cells grown on the scCO_2_-treated porous silicon, compared to cells grown on the untreated or plasma-treated porous silicon. In addition, the expression of several apoptosis genes were suppressed, relative to the untreated or plasma-treated surfaces.

## 1. Introduction

Porous silicon has been employed in biomedical applications, including in vivo studies in the human body for therapy and diagnostics, and in vitro for biosensing and biofiltration [1]. Porous silicon can cause inflammation; its biocompatibility when implanted is related to its pore size [2]. Porous silicon particles have been used as carriers for drug delivery, exploiting their porosity to encapsulate bioactive molecules [3]. Porous silicon has also been employed in biosensing devices, utilizing optical properties including photoluminescence, thin-film reflectance, and photonic effects [4]. The high surface area of porous silicon can offer more binding sites for the immobilization of recognition elements than could be obtained with flat (smooth) surfaces [5,6].

Recently, nanomaterials used in cardiac tissue engineering have been reviewed by Kankala et al. [7]. That review noted that mesoporous silica nanoparticles have some attractive physicochemical features, such as well-defined structure, tunable pore morphology, high surface area and drug loading ability, biocompatibility, biodegradability, colloidal stability, and high dispersity [7]. “Biocompatible” is a relative term, however: for example, neural stem cells grown on silicon carbide showed less oxidative stress and fewer morphological modifications or adverse reactions in mitochondrial membrane potential, compared with cells grown on silicon [8]. Moreover, long-lived, transferred crystalline silicon carbide nanomembranes were demonstrated for implantable flexible electronics [9], showing the potential in vivo uses of silicon carbide materials. Thus, surface modification of pSi to further improve its biocompatibility is warranted.

In this study, silicon electrochemically etched with HF underwent further surface modification using either plasma or supercritical carbon dioxide (scCO_2_). The morphologies and compositions of the porous silicon were then examined by scanning electron microscopy (SEM) and electron spectroscopy for chemical analysis (ESCA). The specific surface areas of these porous structures were obtained by fitting the adsorption and desorption of nitrogen using Brunauer–Emmett–Teller (BET) analysis. The contact angles of these porous structures were also measured to determine their hydrophobicities. Following these physicochemical characterizations, H9c2 cardiomyocytes were grown on the silicon substrates and their viability was assessed using the 3-(4,5-Dimethylthiazol-2-yl)-2,5-diphenyltetrazolium bromide (MTT), live/dead™ viability/cytotoxicity assays, and 4′,6-diamidino-2-phenylindole (DAPI) staining. Finally, the expression of angiogenesis and apoptosis genes was investigated using real-time quantitative reverse transcription polymerase chain reaction (qRT-PCR).

## 2. Results and Discussion

**Figure 1:**
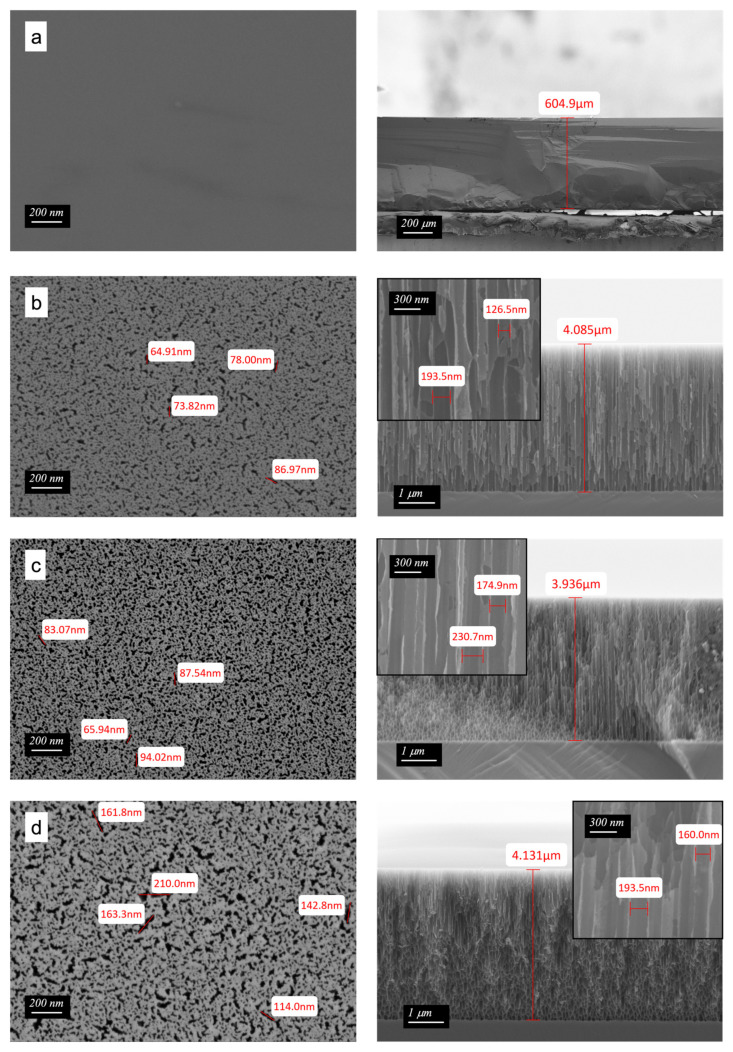
FE-SEM images of the (**a**) silicon substrate (S1), (**b**) after etching (S2), (**c**) O_2_ plasma (S3), or (**d**) scCO_2_ treatment (S4). Images in the right column are the cross sections of the samples, and the insets are the enlargements of samples after etching.

Figure 1 presents the surface morphologies and cross-sections of silicon substrate (sample S1), after etching (sample S2) and after surface modification by treatment with O_2_ plasma (sample S3) or scCO_2_ (sample S4). The unmodified wafer shows no porosity. Electrochemical etching with HF created many pores to a depth of about 4 μm. Pore size is highly heterogeneous; the largest pores are elongated with a long dimension of approximately 80–90 nm. Figure 1b,c show how the porosity increased as a result of plasma treatment. Interestingly, the sizes of the largest pores did not increase in size. Rather, the most notable effect of plasma treatment was to increase the sizes of the small pores. Figure 1d shows the surface of an HF-etched wafer subsequently treated with scCO_2_. All of the pores were enlarged by the scCO_2_ treatment, a somewhat surprising result that likely results from the action of residual HF. The cross-sections of the porous silicon in the right-hand column of Figure 1 show that the thickness of the porous structures was approximately 4 μm.

**Figure 2:**
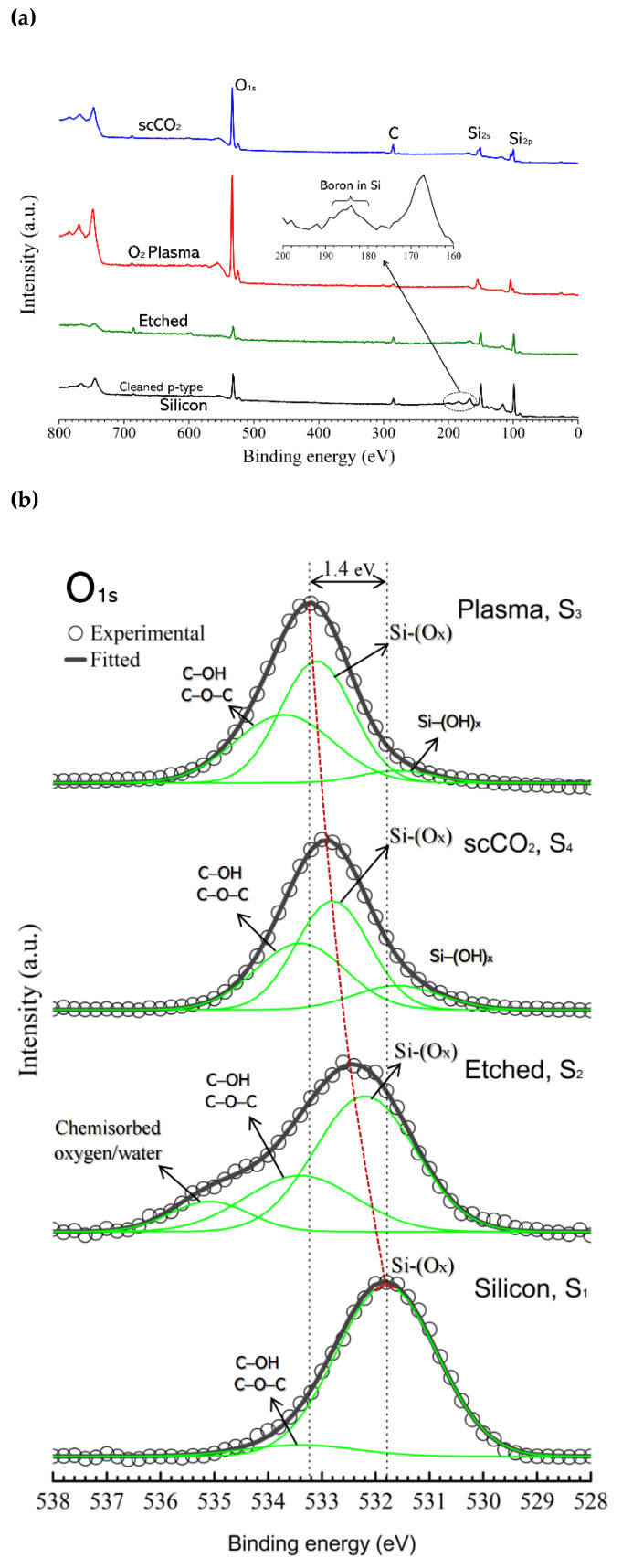
(**a**) XPS wide-scan spectra for cleaned p-type silicon and after etching and treatment with O_2_ plasma or scCO_2_. (**b**) Deconvolution of XPS spectra of O_1s_ for different samples.

Figure 2a presents the results of wide-scan electron spectroscopy for chemical analysis/X-ray photoelectron spectroscopy (ESCA/XPS) for cleaned p-type (boron-doped) silicon substrate (sample S1), freshly etched pSi (sample S2) (i.e., less than 2 days after etching) and pSi treated with O_2_ plasma (sample S3) or scCO_2_ (sample S4). It shows the atomic contents of these samples, including silicon, carbon and oxygen; the peaks at ~100, 150, 285 and 532 eV correspond to states of Si_2p_, Si_2s_, C_1s_ and O_1s_, respectively. High-resolution XPS spectra of Si_2p_, C_1s_ and O_1s_ are shown in the Figure S1a–d, Figure S1e–h and Figure 2b, respectively for samples S1–S4. All the binding energies (BE) are referenced to the C_1s_ peak at 285 eV and given with a precision of 0.2 eV. Table 1 presents the reported XPS peak binding energies and the corresponding chemical structures for Si_2p_, C_1s_ and O_1s_, respectively. Using multiple Gaussian peaks for fitting and the adopted peaks selected from Table 1, the relevant chemical states for each sample were identified and are indicated in Figure 2, and also in Table S1. In Table S1, the total atomic contents expressed in atomic percentage (%, Total O, C and Si) for different samples are shown, as determined from the individual integrated areas of O_1s_, C_1s_ and Si_2p_ XPS spectra divided by the total area (O_1s_ + C_1s_ + Si_2p_), using JEOL SpecSurf version 1.9.5 software and considering the atomic Scofield sensitivity factor. Figure S2 shows the composition ratio of C/Si and O/Si for different samples; the bars in Figure S2 give the surface composition %, as shown in Table S1. The high oxygen and low carbon contents in the plasma-treated sample are noteworthy. The scCO_2_-treated sample had somewhat higher oxygen and carbon contents, compared with the etched or virgin wafer.

**Figure 3:**
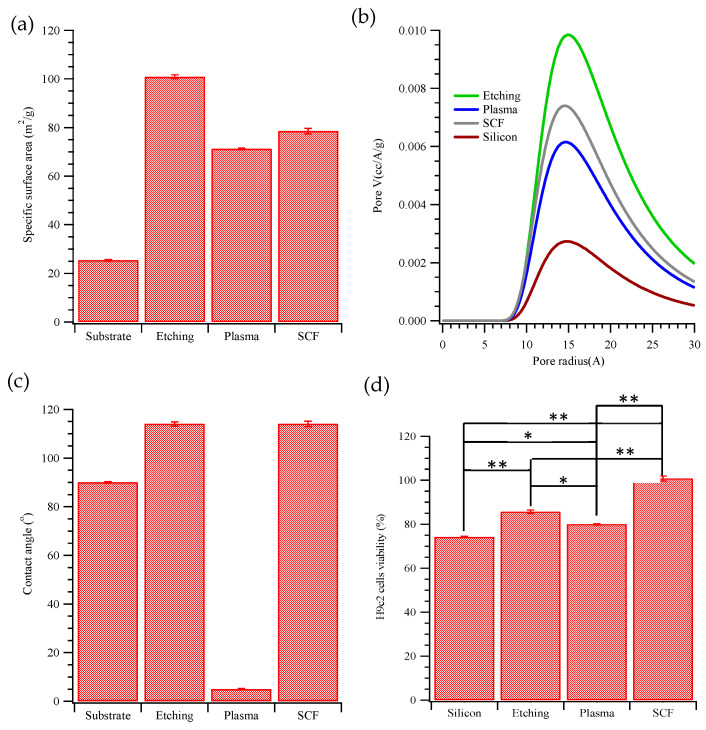
(**a**) The specific surface areas measured by nitrogen adsorption, (**b**) pore size distribution fitted by the Barrett-Joyner-Halenda (BJH) model, (**c**) the contact angles, and (**d**) viability (using the MTT test) of H9c2 cells grown on various substrates: tissue culture polystyrene (TCPS), silicon substrate, after etching, plasma treated, or scCO_2_ treated (* *p* < 0.05, ** *p* < 0.001).

Figure S3 and Figure 3a,b display the nitrogen gas adsorption/desorption curves, specific surface areas and pore size distribution curves, respectively. In Figure S3, based on the IUPAC classification of hysteresis loops [24], all samples exhibit H3 loops. The slit-shaped pores in porous silicon are consistent with the SEM images in Figure 1. In Figure 3a, the specific surface areas of the silicon substrate before etching, after etching, after plasma, and after scCO_2_ treatment are 25.4, 100.8, 71.2 and 78.5 m^2^/g, respectively. The pore size distribution fit the Barrett-Joyner-Halenda (BJH) model; the mean pore radii were 15–16 Å, calculated using the Horvath-Kawazoe (HK) model, as shown in Figure 3b. Figure 3c presents the water contact angles on the silicon substrate after etching, plasma, and scCO_2_ treatment. Both the etched silicon and the scCO_2_-treated etched silicon were hydrophobic, with water contact angles from 90–114°. In contrast, the etched silicon substrates that were treated with oxygen plasma had a very hydrophilic surface with a contact angle of around 5°.

The principal aim of this work was to study the effects of the novel scCO_2_-treated surface on cell viability and behavior, comparing the results with those obtained on etched and plasma-treated etched surfaces. Cell viability and mitochondrial membrane potential (a measure of oxidative stress) were measured using optical absorption and fluorescence assays; to avoid difficulties with these measurements on an opaque and semiconducting substrate, after 1 day of growth on pSi, cells were removed and transferred to tissue culture polystyrene (TCPS) and grown for an additional 24 h. (Experimental details can be found in the supplemental material.) Figure 3d presents the viability of H9c2 cardiomyocytes incubated on silicon substrates after etching, plasma, or scCO_2_ treatment.

**Figure 4:**
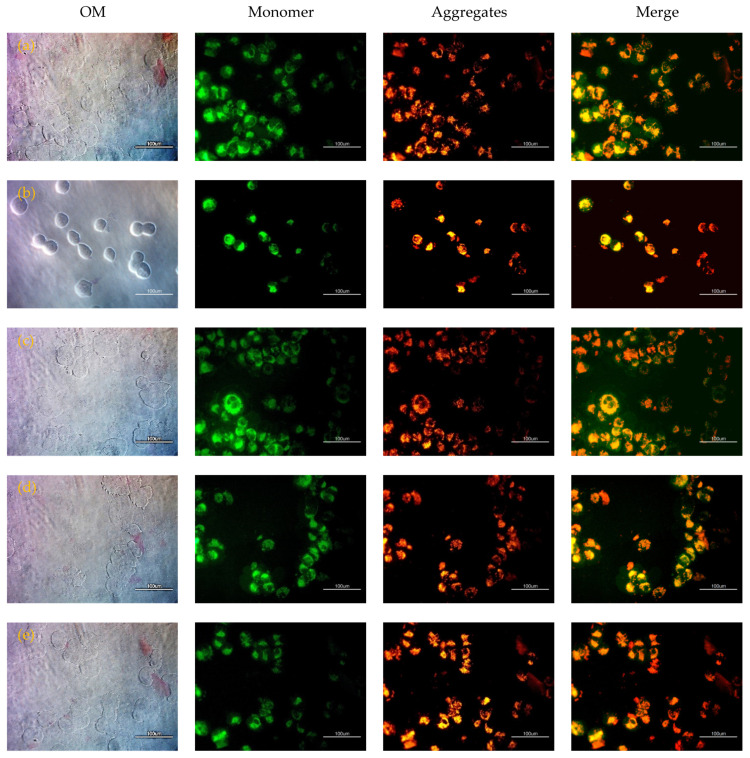
Optical imaging and JC-1 staining of H9c2 cells grown on various substrates: (**a**) TCPS, (**b**) silicon substrate, and (**c**) after etching, (**d**) plasma, or (**e**) scCO_2_ treated. (Scale bar = 100 μm).

The viabilities were normalized to that of the control cells that had not been exposed to porous silicon. Both the etched and the etched and plasma-treated surfaces gave slightly reduced viability—about 74.3 ± 0.3~85.7 ± 0.8% of controls. In contrast, the viability of the H9c2 cells on the silicon (i.e., 100.8 ± 1.1%) that was treated with scCO_2_ was as high as that of the controls. Figure 4 displays staining with JC-1 dye to obtain the mitochondrial membrane potential of H9c2 cells on porous layers. JC-1 partitions between monomeric and aggregated forms, depending on membrane potential. A ratiometric measurement of monomer and aggregate emission thus provides a measure of oxidative stress that is independent of cell number or dye concentration. From top to bottom, Figure 4a–e show fluorescence images of H9c2 cells grown only on tissue culture polystyrene (TCPS, controls), cells grown for 24 h on the silicon substrate before etching, after etching, after plasma treatment, or after scCO_2_ treatment (followed by growth on TCPS for 24 h). Proper mitochondrial membrane potential (i.e., absence of oxidative stress) is indicated by a high concentration of red (aggregate) fluorescence, compared to green, monomer fluorescence (Figure S4). The most poorly performing substrates are the unetched silicon wafer and, interestingly, the plasma-treated, etched substrate. The plasma treatment seems to be deleterious to mitochondrial health. The scCO_2_-treated and untreated etched substrates both gave little oxidative stress, comparable to (or perhaps slightly higher than) the controls The high level of oxidative stress on silicon is consistent with a recent study using an in vitro model of human neuronal stem cells and mouse olfactory ensheathing cells [8].

**Figure 5:**
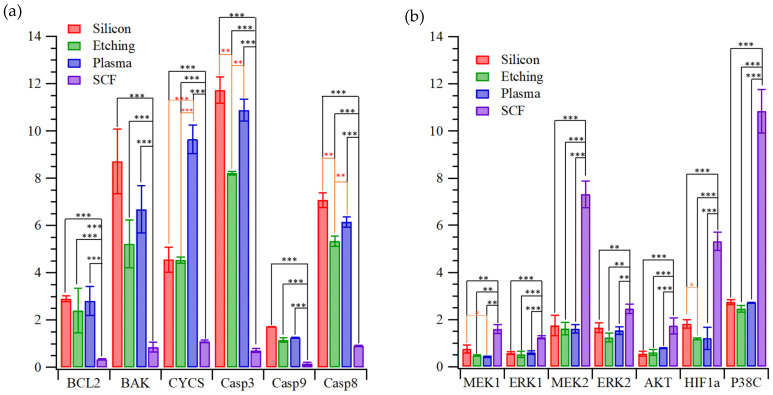
Expression levels (relative to TCPS controls) of key factors in (**a**) apoptosis or (**b**) angiogenesis pathways of H9c2 cells grown on the silicon substrate, after etching, plasma treated, or scCO_2_ treated (* *p* < 0.05, ** *p* < 0.001, *** *p* < 0.0005).

Figure 5 shows gene expression in H9c2 cardiomyocytes cultured on the porous silicon substrates, including genes (Table S2) for apoptosis and angiogenesis (Scheme S1). Figure 5a and Scheme S1a show the high levels of apoptotic gene expression for cells cultured on unetched silicon and plasma-treated etched silicon. These substrates also gave the highest oxidative stress, as was shown in Figure 5. Etched silicon performed slightly better, with somewhat lower levels of apoptotic gene expression, but most remarkable is the very low levels of apoptotic gene expression obtained with the scCO_2_-treated, etched silicon substrate. Angiogenesis is mainly associated with high expressions of P38, HIF1α, Akt MEK1/2 or ERK1/2, Scheme S1b. Importantly, the expressions of MEK2, HIF1α and P38C in Figure 5b are much higher on the scCO_2_-treated porous silicon surface than the other surfaces; thus, this substrate is likely to induce in vitro angiogenesis by cardiomyocytes.

## 3. Materials and Methods

### 3.1. Preparation of Silicon Wafer, after Etching, and Plasma or scCO_2_ Treatments

Electrochemical etching using hydrofluoric (HF) acid-based electrolytes is the most popular method used to fabricate PSi from silicon wafers [25]. In this study, a commercial electrochemical etching cell (The Standard Etch Cell, redoxme AB, Norrköping, Sweden) was used for porosification of silicon wafers. The wafer is the anode, connected on its backside to a copper metal plate; floating graphite electrodes were used as the cathode. A single-sided polished p-type (100) wafer with ρ = 0.02 Ω·cm was purchased form Wafer Work Co. (Taiwan) as the substrate to produce PSi samples. Before etching, the substrates were cleaned in an ultrasonicater with acetone (Taiwan Maxwave Co., Ltd., Taipei, Taiwan), isopropanol (IPA, Honeywell Burdick & Jackson^®^, Seoul, Korea) and deionized (DI) water (Lotun Technic Co., Ltd., Taiwan), successively. Then, an HF/NH_4_F (7.23/34.1, wt%) (Taiwan Maxwave Co., Ltd., Taipei, Taiwan) solution was applied for 5 min to remove the surface native oxidation layer and impurities. After removal of the native oxide, the wafer was immediately introduced into electrochemical anodic etching cell. The HF-based etchant was prepared by mixing 49 wt% aqueous-HF (High Standard Enterprise Co., LTD., Taichung, Taiwan), 95 vol% ethanol (Ritai chemical, Kaohsiung, Taiwan) and DI water in the volume ratio of 12:12:33, which gives a concentration of [HF] ~12 wt%. 30 mA of constant current (Model 2401, Keithley Instruments Inc., Solon, Ohio, USA) was applied, giving an estimated current density of ~25 mA/cm^2^ based on the exposed wafer area. The etching time was fixed at 400 s, delivering 12 C of charge. The etched PSi samples were then gently rinsed in ethanol, dried with N_2_ gas and kept on the hot plate at 70 °C for 10 min. Finally, the etched PSi samples were transferred into a vacuum box for vacuum drying for 10 min. Etched PSi samples were then separately treated by O_2_ plasma and scCO_2_. O_2_ plasma treatment was carried out using a high power expanded plasma cleaner (Model: PDC-001-HP, Harrick Plasma, Ithaca, NY, USA) in vacuum of ~170 mTorr, using radio frequency power of 45 W and 20 standard cubic cm per minute (sccm) O_2_ flow for 3 min. For scCO_2_ treatment, pSi samples were processed in a 300 mL stainless steel pressure vessel. CO_2_ was added to the vessel to a pressure of ~800 psi in 10 min; then the temperature was increased from room temperature to 140 °C in 1 h (raising the pressure to about 1000 psi.) Additional CO_2_ was added and further heating brought the vessel to 3000 psi and 150 °C, which was maintained for 3 h. After complete reaction, the pressure was released in 1 h to the atmosphere. 

Experimental details on cell preparation and staining are provided in the supplemental materials. 

### 3.2. Characterization of Silicon Wafers after Etching, and Plasma or scCO_2_ Treatments

Silicon wafers after etching, and after plasma or scCO_2_ treatments, were vacuum dried before examination by a scanning electron microscope (Hitachi S4800, Hitachi High-Technologies Co., Tokyo, Japan). Electron spectroscopy for chemical analysis (ESCA, PHI 5000 VersaProbe/Scanning ESCA Microprobe, ULVAC-PHI Inc., Chigasaki, Kanagawa, Japan) was employed to measure the elemental composition of the silicon wafer after etching, and after plasma or scCO_2_ treatments. Nitrogen adsorption measurements were performed with a NOVA 1000e, and Brunauer–Emmett–Teller (BET) analysis was performed with the Autosorb program (Quantachrome Instruments, FL, USA). BET analysis was carried out with nitrogen gas at relative vapor pressure of 0.05–0.3 at 77 K. The contact angles of water on these samples were measured on a contact angle goniometer (Model 100, Sindatek Instruments Co., Ltd., Taipei, Taiwan) according to the sessile drop method.

### 3.3. Data Analysis

All experiments were carried out in triplicate, and data are expressed as means ± standard deviation. The data was analyzed with Student’s *t*-test. Statistical significance was set at a *p*-value of less 0.05, highly significant as *p* < 0.001, and dramatically significant as *p* < 0.0005.

## 4. Conclusions

Porous silicon has relatively high biocompatibility, but also may lead to the generation of reactive oxygen species (ROS) [26], which may reduce that biocompatibility through oxidative stress. This study has shown that post-etch treatment of porous silicon with supercritical CO_2_ not only improves biocompatibility, but also reduces the expression of some apoptosis genes and enhances the expression of some angiogenesis genes. At this stage, it is not clear whether these beneficial changes are a consequence of the slightly altered morphology or of the changes in surface chemistry caused by the scCO_2_ treatment. scCO_2_ causes a slight enlargement of the pores, particularly the smaller pores, most likely through the action of residual HF. scCO_2_ treatment also changes the surface physical chemistry, rendering it hydrophobic (compared to plasma-treated, etched porous silicon) and altering the surface composition (unsurprisingly, increasing carbon and oxygen contents.) Regardless of the mechanism of action, the result is quite promising for the future use of scCO_2_-treated porous silicon for cardiac tissue engineering.

## Figures and Tables

**Table 1:** Chemical structure and binding energy (EB) present in the samples.

		State	Binding Energy (EB) [eV]			
**Si2p**						
**Metallic Silicon**			98.4 [10,11,12,13]			
p-type Si		Si0 3/2	99.2 [10]			
Si–(Si_4_)		Si0 3/2	99.4 [14]	99.5 [15]	99.6 [16]	99.6 [17]
		Si0 1/2	100.0 [14]	100.0 [15]	100.5 [16]	100.2 [17]
	Si–(Si_3_O)	Si^+^	100.4 [14]	101.0 [15]	100.4 [16]	100.6 [17]
	Si–(Si_2_O_2_)	Si^2+^	101.4 [14]	101.5 [15]	101.9 [16]	101.4 [17]
	Si–(SiO_3_)	Si^3+^	EB_3/2_: 102.5 [14] EB_1/2_: 103.1 [14]	102.5 [15]	102.6 [16]	102.1 [17]
	Si–(O_4_)	Si^4+^	EB_3/2_: 103.6 [14] EB_1/2_: 104.2 [14]	103.6 [15]	103.7 [16]	103.5 [17]
	Si–O–C		102.2~102.5 [18]			
	Si–O–R		102.3 [16]			
	Si–R		101.8 [16]			
	Si–C		101.3 [16]			
(CH_3_CH_2_)_3_SiOH			101.3 [19]			
					
**C1s**						
C=C			284.5 [14,16]			
C–C, C–H			285 [20]	284.9 [21]	285.1 [16]	
C–O–C, C–OH			286.6 ± 0.2 [20]	286.7 [21]	286.5 [14]	
O–C–O, O–C=O			288.2 ± 0.2 [20]	288.8 [21]		
C–Si			283.9 [16]			
Carbide			283.7 [22]	282.8~283.6 (B_4_C) [21]		
			
**O1s**						
Si–(OH)_x_			531.1 [23]@Si			
Si–(O_2_)			531.9 [23]@Si			
Si–(O_4_), SiO_2_			532.9 [23]@Si	532.5~533.1 [14]		
C–OH, C–O–C			~533.4 [14]			
Chemisorbed Oxygen & Water			534.6~535.4 [14]			

## Data Availability

The authors confirm that the data supporting the findings of this study are available within the article and its supplementary materials.

## Supplementary Materials

The supplementary materials are available online at https://www.mdpi.com/article/10.3390/ijms221910709/s1.
