# Supplementary material for: Supercritical Carbon Dioxide Treatment of Porous Silicon Increases Biocompatibility with Cardiomyocytes
Source: Int J Mol Sci. 2021 Oct 2;22(19):10709. doi: 10.3390/ijms221910709 (PMC8509595; doi:10.3390/ijms221910709)

Supporting inforamtion:

Supercritical Carbon Dioxide Treatment of Porous Silicon Increases Biocompatibility with Cardiomyocytes

David Jui-Yang Feng[1,], Hung-Yin Lin[2], James L. Thomas[3], Hsing-Yu Wang[1], Chien-Yu Lin[2], Chen-Yuan Chen[2], Kai-Hsi Liu[1,4], and Mei-Hwa Lee[5,*]*

[1]Department of Electrical Engineering, National University of Kaohsiung, Kaohsiung 81148, Taiwan

[2]Department of Chemical and Materials Engineering, National University of Kaohsiung, Kaohsiung 81148, Taiwan

[3]Department of Physics and Astronomy, University of New Mexico, Albuquerque, NM 87131, USA

[4]Department of Internal Medicine, Division of Cardiology, Zuoying Branch of Kaohsiung Armed Forces General Hospital, Kaohsiung 81342, Taiwan

[5]Department of Materials Science and Engineering, I-Shou University, Kaohsiung 84001, Taiwan

* To whom correspondence should be addressed:
(D. J.-Y. Feng) Department of Electrical Engineering,
National University of Kaohsiung (NUK),
700, Kaohsiung University Rd.,
Nan-Tzu District, Kaohsiung 81148, Taiwan
Tel: (O) +886(7)591-6586; (M) +886(918)136-069
E-Mail: djyfeng@nuk.edu.tw

(M.-H. Lee)Department of Materials Science and Engineering,
I-Shou University,
No.1, Sec. 1, Syuecheng Rd., Dashu District,
Kaohsiung City 84001, Taiwan.
Tel./fax: +886 (7) 657 8228
E-Mail: meihwalee@ntu.edu.tw

S1 Experimental

S1.1 Cell viability and Histochemistry/JC-1 staining

The H9c2 cardiomyocyte cell line from rat heart myoblast was purchased from the Bioresource Collection and Research Center (BCRC; Taipei, Taiwan). The H9c2 cells (1×10^4 cells) were seeded onto the porous silicon samples (0.4x 0.4 cm^2) in 24-well plates in DMEM/F12 medium at 37 °C and 5% CO_2 for 24 h. After 24 h, the cells were trypsinized, gently washed and replated into a new 24-well plate (without silicon) and grown for an additional 24 h prior to MTT and JC-1 optical studies. (This procedure was employed because 1) the MTT test requires measurement of absorption, and the opaque substrate would prevent this; and 2) direct fluorescence microscopy on the silicon substrate was not feasible; moreover, the semiconducting silicon substrate could in principle affect fluorescence measurements.) . (1) The MTT test was used for assessing the viability of cells after exposure; briefly, MTT solution (10 μL) was added to each well and incubated for 3 h, followed by removal of media and addition of 100 μL DMSO to dissolve the formazan crystals. Absorbance at wavelengths of 570 nm and 690 nm was measured with a plate reader (CLARIO star, BMG LABTECH, Germany). Cell viability (%) was calculated as a percentage with respect to untreated cells. (2) DAPI fluorescence. MEM was removed and then cells were washed with phosphate buffered saline (PBS) three times in each well. Cells were fixed with 3.7% paraformaldehyde (PFA) for 10 min. After removing PFA, cells were washed with PBS three times. 0.1% Triton-X-100/PBS

was used to permeabilize cells for 5 minutes. This solution was removed, and then PBS was added and cells were washed three times. Bovine serum albumin in PBS at 7.5% was added and incubated at 4 °C for 1 h. The bovine serum albumin solution was removed, and then washed with PBS three times. DAPI at 1 μL/mL in PBS was added to stain cells for 5 minutes. PBS was added and cells washed three times after DAPI was removed. The 24-well culture plate was placed in the dark at room temperature until each well was dry. The morphologies of cell nuclei were obtained using an inverted fluorescence microscope (CKX41, Olympus, Melville, NY). (3) For JC-1 staining, culture medium was removed and cells were washed with 0.5 mL PBS for each well. The 400 μL JC-1 solution was added and cells were incubated for 20 minutes at 37 °C. The JC-1 agent was removed and the cells were washed twice with DMEM medium. The stained cells were observed by fluorescence microscopy and analyzed on a fluorescent microplate reader (CLARIO star, BMG LABTECH, Germany). The excitation / emission wavelength are 525 / 590 and 490 / 530 nm for JC-1 aggregates and monomer, respectively.

Electron microscopy of H9c2 cardiomyocytes: samples were immersed in (1) 2.5 % gluteradehyde at 4 °C for 2 h; (2) 30, 50, 70, 80, 90 and 100 % ethanol solutions for 10 to 15 min; (3) acetone 15 min, twice, for dehydration. Samples were freeze-dried overnight and coated with Au and then examined in the electron microscope.

S1.2 Gene Expression of Cardiomyocytes Cultured on Silicon wafers, after etching, and after plasma or $scCO_2$ treatments

The sequence (5'- 3') of primers for GAPDH, BCl-2, BAK, CYCS, Casp9, Casp3, Casp8, MEK1, MEK2, ERK1, ERK2, AKT1, AKT2, HIF1-α, and P38C are listed in Table S2. H9c2 cardiomyocytes were centrifuged at 1000 rpm for 5 min to remove culture medium. The total RNA extraction was performed using the Nucleospin RNA, Mini kit for RNA purification (740955.50 Macherey-Nagel). Complementary DNA was obtained following a Magic RT Mastermix cDNA synthesis kit (BB-DBU-RT-100, Bio-genesis Technologies, lnc., Taiwan) protocol. The real-time PCR was then performed with IQ2 SYBR Green Fast qPCR System Master Mix (BB-DBU-006-5, Bio-genesis Technologies, lnc., Taiwan) in a StepOne™ Real-Time PCR System (LS4376357, Applied Biosystems, Waltham, MA). Relative gene expression was determined using a ΔΔCq method and normalized to a reference gene (GAPDH) and to controls (H9c2 cardiomyocytes grown on tissue culture polystyrene (TCPS)).

Table S1. Composition of the samples determined by XPS expressed as atomic %.

Unit：%		Composition	Silicon (S_1)		Etched (S_2)		Plasma (S_3)		$scCO_2$ (S_4)	
**Total O_{1s}**			**21.3**		**16.9**		**61**		**42.7**	
		Si-(O_x)	93.1		61.4		53.7		49.9	
		Si-$(OH)_x$	6.9		0		5.5		13.1	
		C-OH, C-O-C	0		27.9		40.8		37.0	
		Chemisorbed O_2/H_2O	0		10.7		0		0	
**Total C_{1s}**			**17.1**		**18.4**		**4.9**		**16.7**	
		Carbide	0		0		17.3		0	
		C-Si	15.9		11.8		15.4		12.3	
		C=C, C-C	62.9		54.3		51.5		62.1	
		C-O-C, C-OH	14.6	21.2	27.9	33.9	12.4	15.8	25.6	25.6
		O-C-O, O-C=O	6.6		6.0		3.4		0	
**Total Si_{2p}**			**61.6**		**64.7**		**34.1**		**40.6**	
Si^0	Si	Si-(Si_4)	96.9		78.9		19.1		59.1	
Si^+	Si_2O	Si-(Si_3O)	3.1		21.1		2.7		4.9	
Si^{2+}	SiO	Si-(Si_2O_2)	0		0		3.5		5.8	
Si^{3+}	Si_2O_3	Si-C, $(CH_3CH_2)_3SiOH$<br>Si-(SiO_3)<br>Si-O-R	0	3.1	0	21.1	8.2	80.9	13.4	40.9
Si^{4+}	SiO_2	Si-(O_4)	0		0		66.4		23.9	

Table S2. The sequence (5'- 3') of primers for GAPDH, BCl-2, BAK, CYCS, Casp9, Casp3, Casp8, MEK1, MEK2, ERK1, ERK2, AKT1, AKT2, HIF1-α, and P38C.

mRNA	Forward/Reverse	Sequence (5'- 3')
GAPDH	forward	CTTTTGCGTCGCCAG
	reverse	TTGATGGCAACAATATCCAC
BCl-2	forward	CCAGAATCAAGTGTTCCGC
	reverse	CCAGAGAAAGAAGAGGAGTTATAAT
BAK	forward	CTATGACTCAGAGTTCCAGAC
	reverse	AATTGATGCCACTCTCAAAC
CYCS	forward	AAGAACAAAGGCATCATCTG
	reverse	GCTATTAAGTCTGCCCTTTC
Casp9	forward	CTCTACTTTCCCAGGTTTTG
	reverse	TTTCACCGAAACAGCATTAG
Casp3	forward	AAAGCACTGGAATGACATC
	reverse	CGCATCAATTCCACAATTTC
Casp8	forward	CTACAGGGTCATGCTCTATC
	reverse	ATTTGGAGATTTCCTCTTGC
MEK1	forward	GATTACATAGTCAACGAGCC
	reverse	CTTCAAATCTGCTCTCTCTG
MEK2	forward	CTGGACTATATTGTGAACGAG
	reverse	CTTGATGAAGGTGTGGTTTG
ERK1	forward	TTCGAACATCAGACCTACTG
	reverse	TAGACATCTCTCATGGCTTC
ERK2	forward	GAAGCATTATCTTGACCAGC
	reverse	TCCATGGCACCTTATTTTTG
AKT1	forward	AAGTACTCTTTCCAGACCC
	reverse	TTCTCCAGCTTGAGGTC
AKT2	forward	CACCATGATGAGGTGAATAC
	reverse	CTACGGAGAAGTTGTTTAAGG
HIF1-α	forward	AAAATCTCATCCAAGAAGCC
	reverse	AATGTTCCAATTCCTACTGC
P38C	forward	AGATTCTGGATTTTGGACTG
	reverse	CCACTGACCAAATATCAACTG

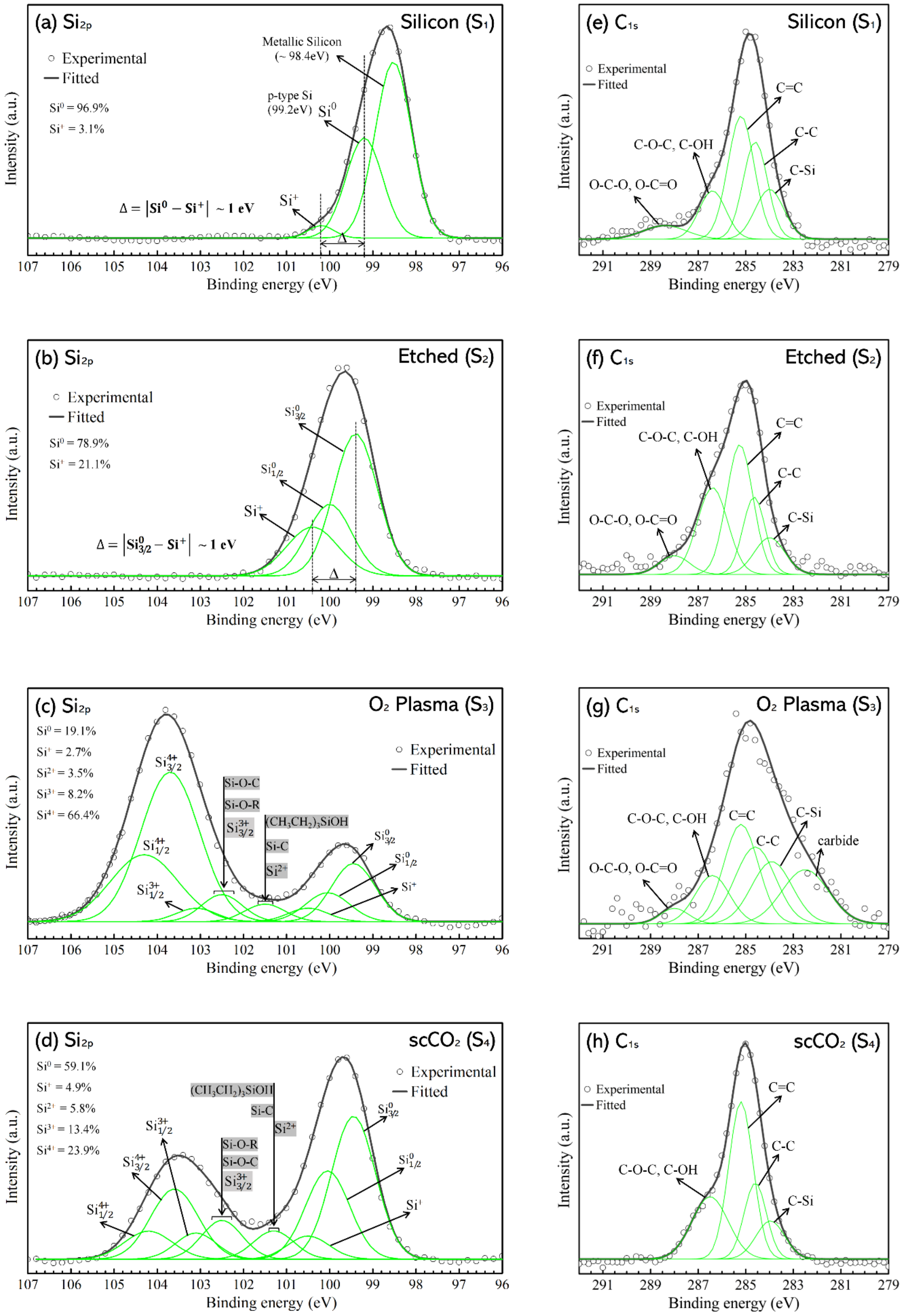

Figure S1. Deconvolved XPS spectra of (a) ~ (d) Si_{2p} and (e) ~ (h) C_{1s} for different samples.

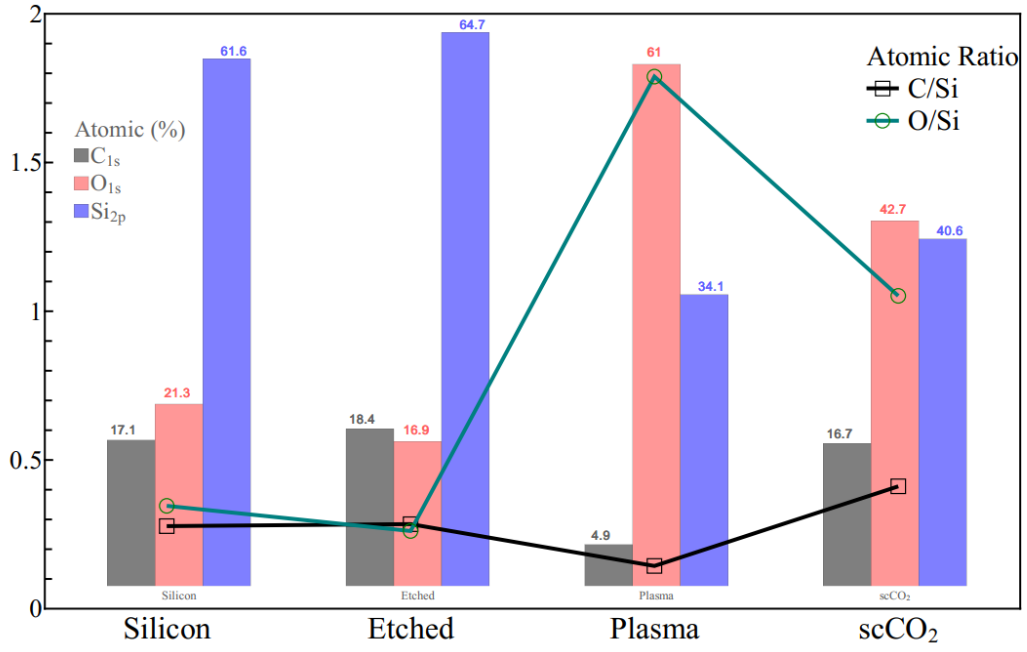

Figure S2. The composition ratio of C/Si and O/Si. The bars show the surface composition %, as in Table S1.

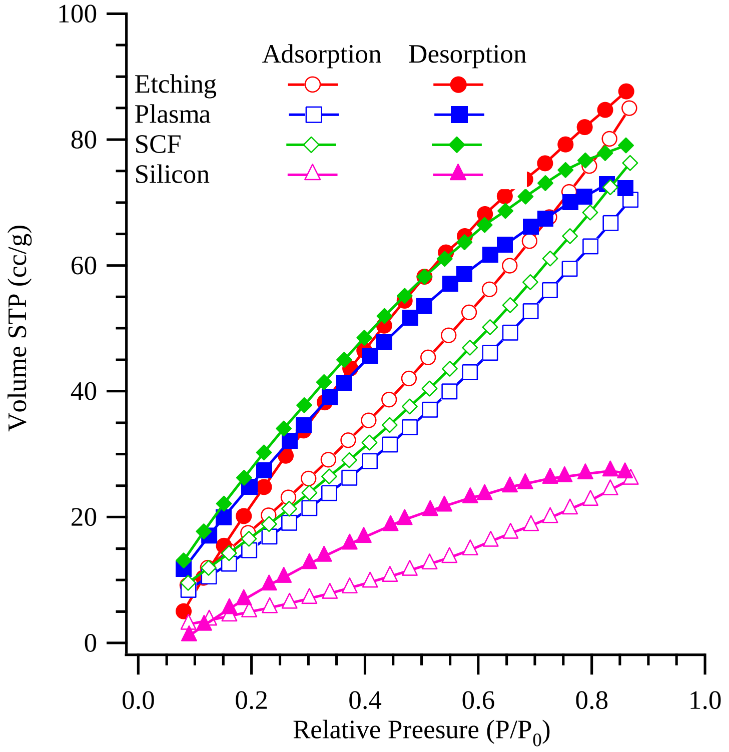

Figure S3. The nitrogen adsorption and desorption on silicon substrate, after etching, and after treatment with plasma, or $scCO_2$.

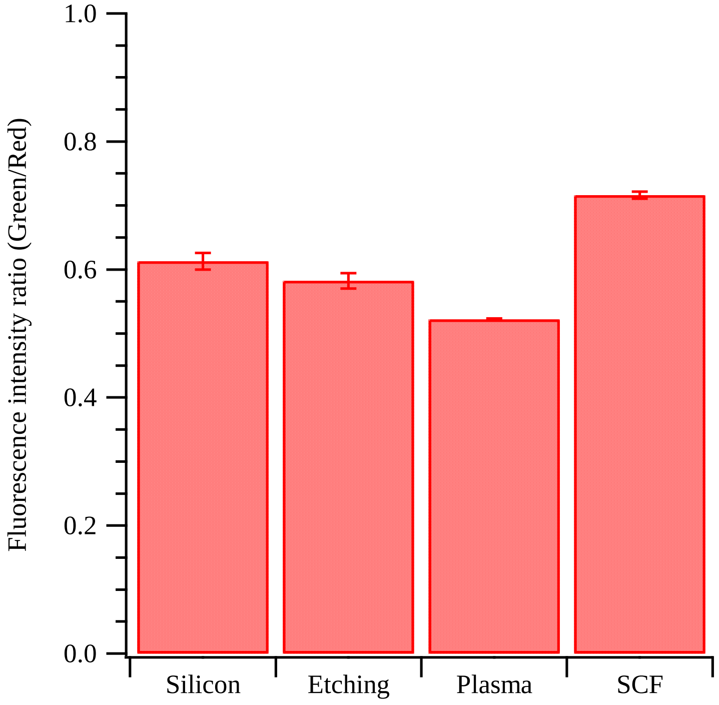

Figure S4. JC-1 florescence intensity ratio of the H9c2 cells on various porous structures calculated from Fig. 4.

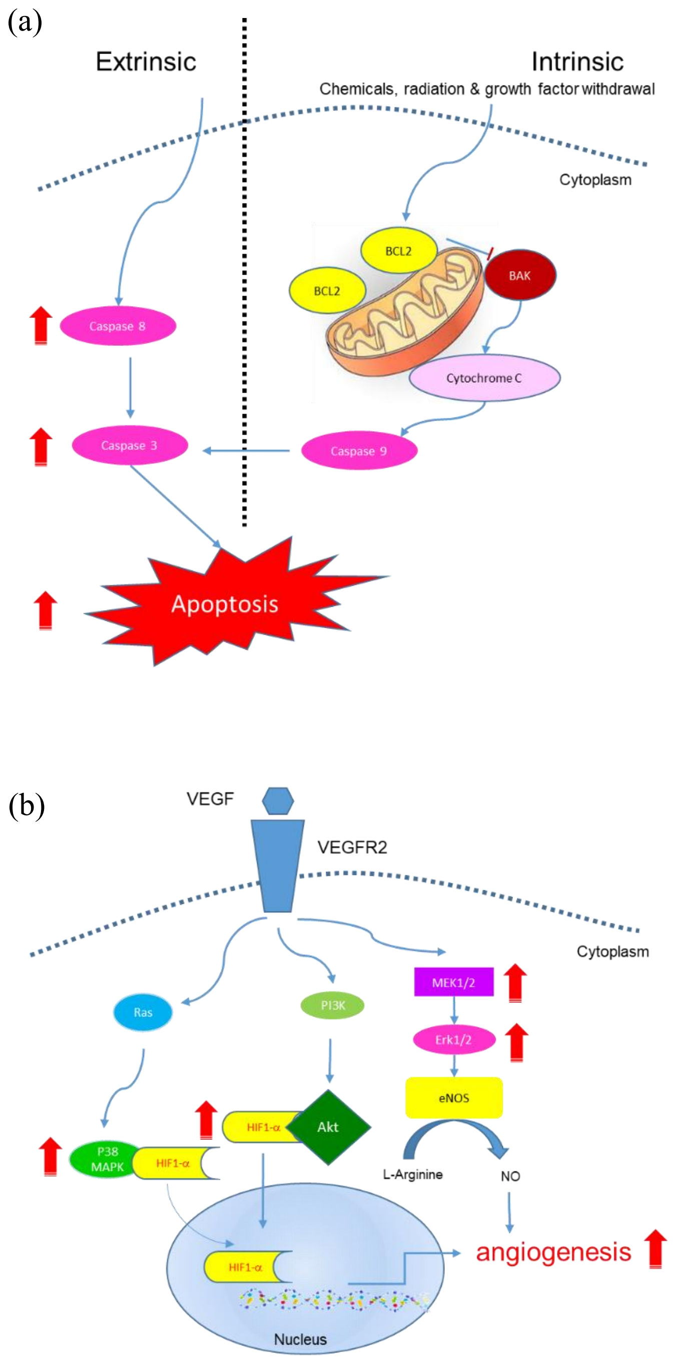

Scheme S1. Signaling of (a) apoptosis or (b) angiogenesis, and arrows indicate the increase of gene expression with incubation of H9c2 cells on porous structure layers.

Graphical Abstract

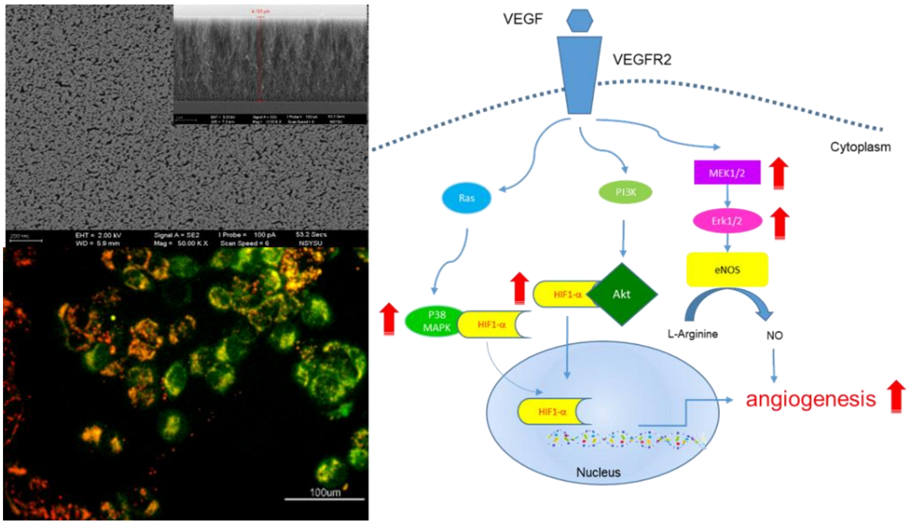

Supplement: Supplementary file 1 [file ijms-22-10709-s001.zip › ijms-1377967-supplementary.pdf]
